# Macular telangiectasia type 2 accompanied by solitary retinal astrocytic hamartoma (case report)

**DOI:** 10.1186/s12886-016-0377-z

**Published:** 2016-11-11

**Authors:** Martin Pencak, Jan Krasny, Miroslav Veith, Magdalena Vokrojova

**Affiliations:** 1 Department of Ophthalmology, University Hospital Kralovske Vinohrady and 3rd Medical Faculty, Srobarova 1150/50, Prague 10, 100 34 Czech Republic; 2 Lexum European Eye Clinic, Antala Staska 1670/80, Prague, Czech Republic

**Keywords:** Astrocytic hamartoma, Macular telangiectasia type 2, Fluorescein angiography (FA), Optical coherence tomography (SD-OCT), Case report

## Abstract

**Background:**

To present a coincidence of macular telangiectasia type 2 and solitary retinal astrocytic hamartoma in one patient.

**Case presentation:**

A 50-year-old woman was examined in the Department of Ophthalmology of University hospital Kralovske Vinohrady for complaints of metamorphopsia in her left eye. Her uncorrected visual acuity (VA) was 4/4 on Early Treatment Diabetic Retinopathy Study charts (ETDRS), on the retina of her left eye white, prominent, partially calcified tumour 1 disc diameter in diameter, 1,5 disc diameter from the foveola was detected on the retina. In the macular region of both eyes, parafoveal greying with crystalline deposits and changes in retinal vasculature were visible. We performed following examinations: fluorescein angiography (FA), B-scan ultrasound, spectral domain optical coherence tomography (SD-OCT) including photo documentation. FA showed partial hyperfluorescence of mulberry-like surface of the tumour typical for retinal astrocytic hamartoma. Parafoveally in both eyes, leakage from parafoveal telangiectasia was apparent. SD-OCT showed cystoid space in the macular region of both eyes as well as changes in inner and outer photoreceptor segment junction in left eye. SD-OCT of the tumour showed proliferation in retinal nerve fibre layer with normal structure of underlying retinal layers and choroid. Ultrasound examination of the tumour detected solid, highly echogenic prominent tumour with high reflectivity and acoustic shadow.

**Conclusion:**

A coincidence of two relatively rare clinical units, macular telangiectasia type 2 and solitary astrocytic hamartoma was detected as a unique and rare observation.

## Background

Retinal astrocytic hamartoma is a benign tumour formed through proliferation of well differentiated astrocytes, mulberry-like based on ophthalmoscopy. It is most common in paediatric patients with tuberous sclerosis or neurofibromatosis [1]. These tumours may be multiple and bilateral in patients with tuberous sclerosis. Both these diseases are classified as the so called phacomatoses, i.e. autosomal dominant diseases with variable penetration and expressivity. Organ symptomatology of phacomatoses (derived from the Greek word "phacoma" or congenital condition; established by Van der Hoeve, Dutch ophthalmologist in 1921) is variegated within the framework of congenital dysplasia of germ layers. In these neuroectodermal forms, it most commonly involves the skin, nervous system and the eye. It is found rarely as unrelated to these diseases, frequently representing an accidental finding in the retina of adult patients [1]. Macular telangiectasia type 2 is a progressive bilateral disease of the macula of unknown etiology, associated with changes in the macular capillary network and retinal atrophy [2]. It may be complicated by the formation of a neovascular membrane and macular hole.

## Case presentation

**Fig. 1:**
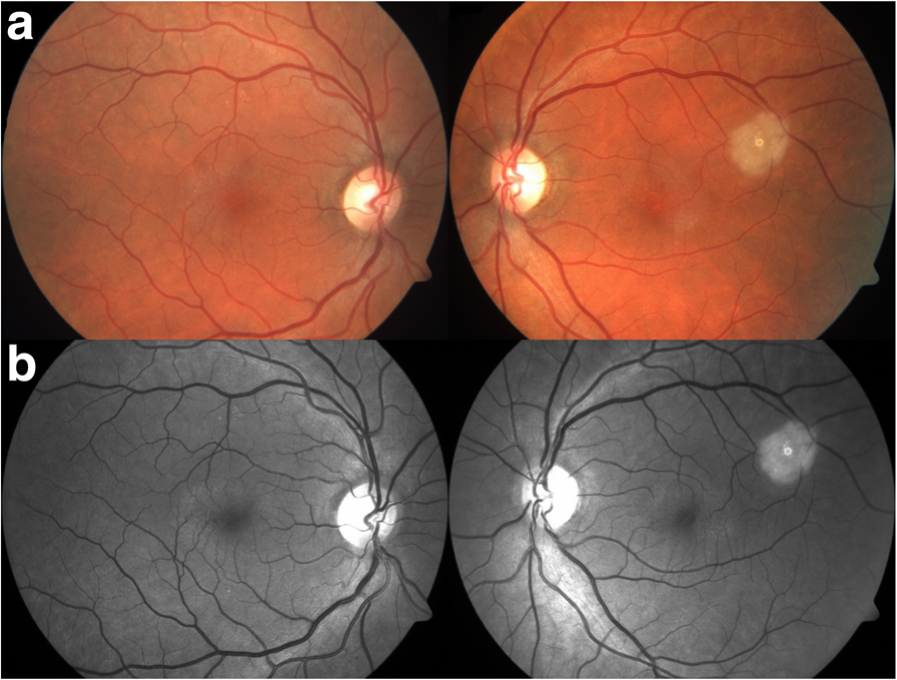
Colour fundus photography (**a**). Whitish tumour with calcification at the centre in the upper temporal region of the left eye. Retinal greying with small crystalline deposits and impaired structure of the capillary network with small telangiectasias in temporal parafoveolar region of both eyes. All changes are better visible in red-free documentation of the fundus (**b**)

**Fig. 2:**
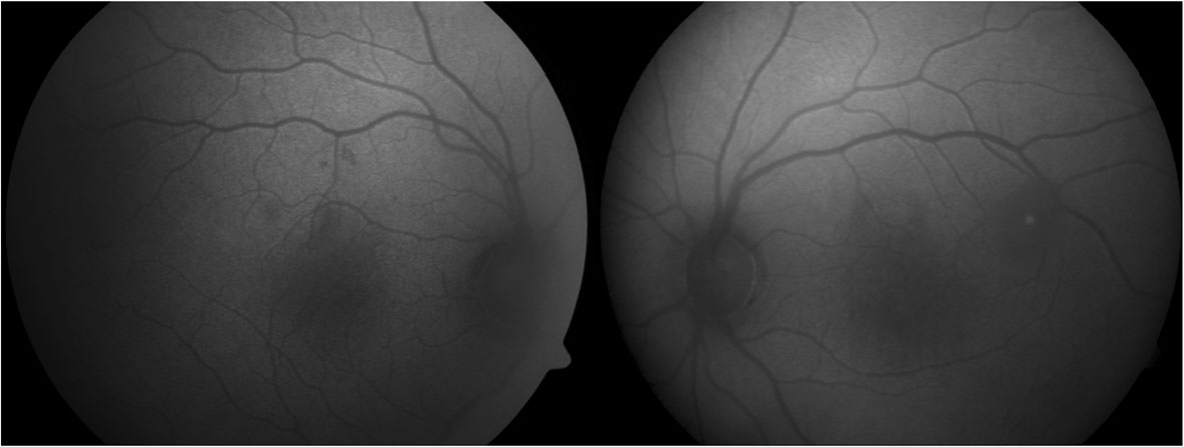
Hypoautofluorescence at the lesion site with hyperautofluorescence of the central calcification in the left eye. Mild increase in autofluorescence in macular regions of both eyes

**Fig. 3:**
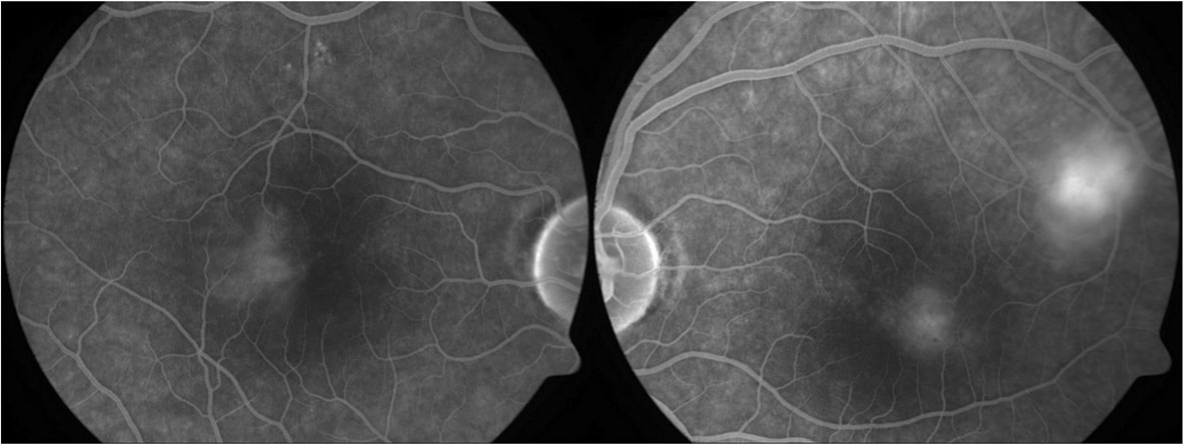
Late phase FA – leakage from parafoveolar telangiectasias in the macula, leakage in the tumour area in the left eye, with accentuation of the mulberry-like structure of the tumour

**Fig. 4:**
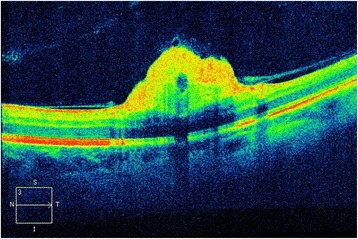
OCT at the tumour site in the left eye – proliferation in the retinal nerve fibre layer with normal structure of deeper layers of the retina and normal choroidea; a shadow at the calcification site

**Fig. 5:**
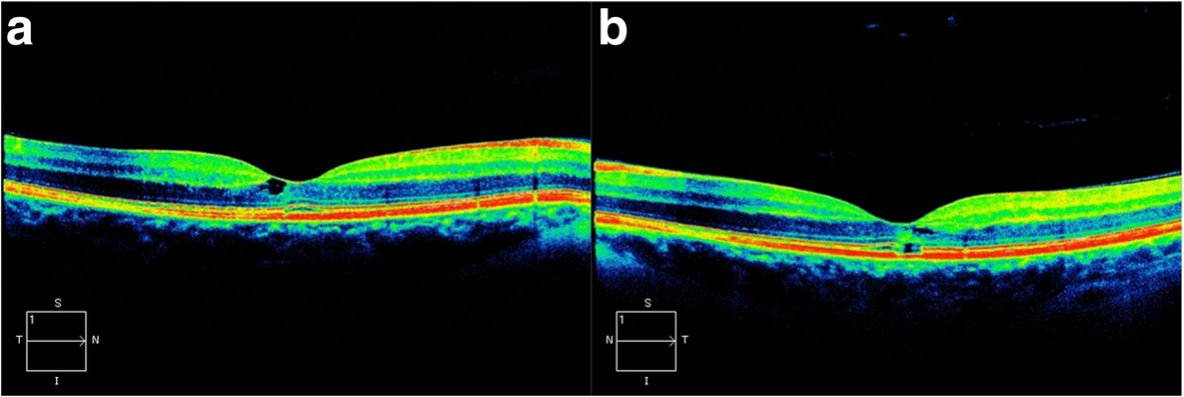
OCT of the macula of the right (**a**) and left (**b**) eye – slight cystic infiltration in both eyes and areas of impairment of retinal architecture at the junction of external and internal photoreceptor segments in the left eye

**Fig. 6:**
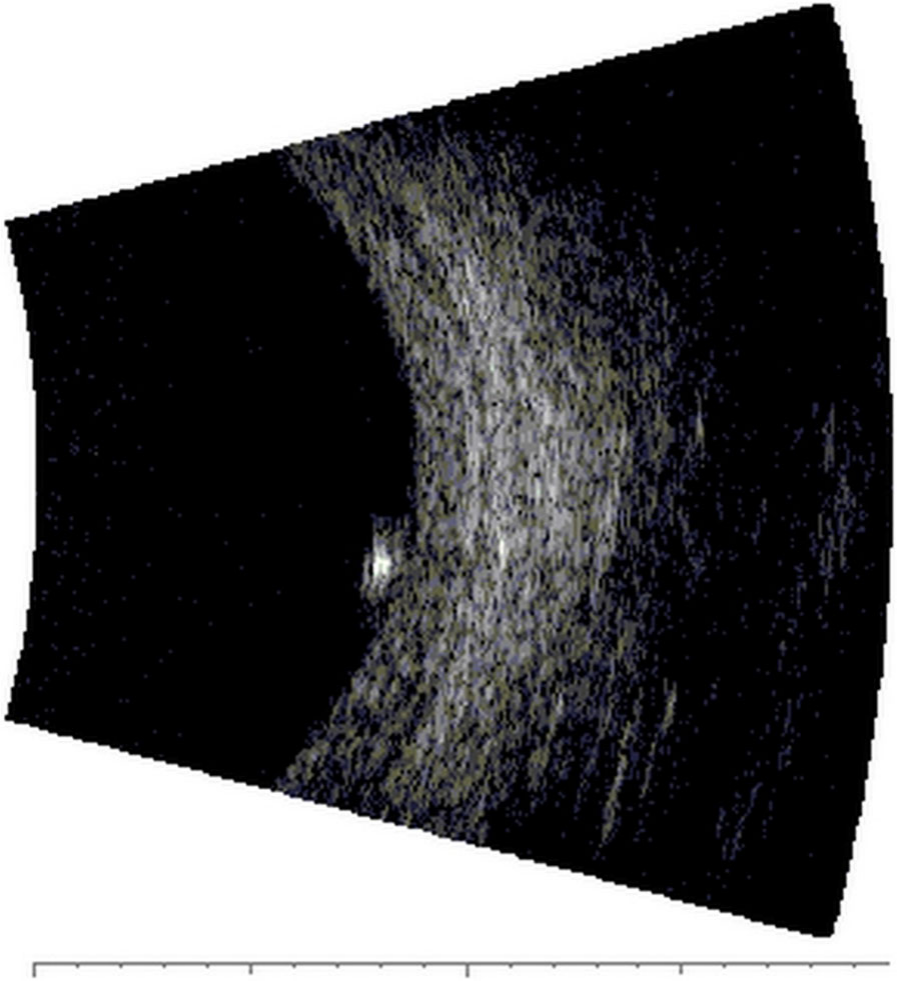
Ultrasound of the left eye – solid, highly echogenic, prominent lesion with high reflectivity and an indicated acoustic shadow

In February 2014, a 50-year-old Caucasian female patient was referred to our department due to suspected chorioretinitis in the left eye. The patient reported metamorphopsia in the left eye lasting about 6 months. Both personal and ocular histories were free of any noteworthy facts. VA was 4/4 (ETDRS) natural in both eyes; intraocular pressure was 26 mmHg in the right eye and 23 mmHg in the left eye. The finding in the anterior segment of both the right and left eyes was quite physiological when examined using the slit lamp. Biomicroscopic examination of the fundus of the left eye revealed a clear, prominent whitish tumour with calcification at the centre (Fig. 1a) sized 1 disc diameter, up in the temporal region at the edge of the macular area, 1.5 disc diameter from the foveola. No supply vasculature or vascular drainage were visible. Furthermore, parafoveal greying of the retina was visible in the parafoveolar temporal regions in both eyes, with small crystalline deposits and impaired capillary network structure with small telangiectasias (Fig. 1a), better visible in red-free photographic documentation (Fig. 1b). Autofluorescence of the fundus in the left eye showed a mild decrease in autofluorescence in the area of the tumour with hyperautofluorescence at the calcification site (Fig. 2). The macular regions of both eyes showed a mild increase in autofluorescence. Fluorescein angiography showed mild fluorescein leakage at the tumour site in the left eye in late phases, with accentuation of the mulberry-like structure of the tumour. In the macular regions of both eyes, the telangiectasias were coloured in early phases with fluorescein leakage in the foveola in late phases (Fig. 3). SD-OCT of the tumour showed proliferation in the retinal nerve fibre layer with a normal structure of deeper retinal and choroid layers (Fig. 4) with centrally located calcification associated with an acoustic shadow. In the area of the foveola with normal contours, small cystoid areas were visible in both eyes, as well as small areas of impaired retinal architecture at the junction of external and internal photoreceptor segments (Fig. 5). Ultrasound assessment of the left eye showed a solid, highly echogenic lesion in the temporal region above the central area, prominent by 0.65 mm, with high reflectivity 85-90 % with central calcification and an indicated acoustic shadow (Fig. 6). The finding was concluded as macular telangiectasia type 2 associated with a solitary retinal astrocytic hamartoma. No skin alterations were shown in the patient, in the sense of neuroectodermal phacomatoses, and her general condition was not associated with neurological symptomatology either. The additionally collected history confirmed no family burden with the above mentioned diseases. The 85-year-old mother of the patient has been followed at our department due to bilateral active neovascular membrane; however, the advanced stage of the finding at the time of diagnosis prevented us from determining whether it had developed in connection with macular telangiectasia of the mother. To confirm our diagnosis and to rule out familial form of macular telangiectasia type 2 we also examined the 47-year-old brother and, with consent of his parents, 7-year-old son of the patient but found no signs of macular telangiectasia or astrocytic hamartoma.

Considering that currently no efficient therapy of macular telangiectasia type 2 is known, and given the stability of the astrocytic hamartoma without any signs of growth after 18 months of follow-up, the patient is only actively followed without any therapy for now.

## Discussion

In most cases, astrocytic hamartomas are benign lesions showing no tendency to grow and cause complications and without any need for a therapeutic intervention. The ophthalmological presentation is very variable ranging from flat to elevated lesions of whitish or yellow colour. They tend to be slightly opaque or transparent, with not very well defined boundaries. Calcifications are common in the lesion. Spontaneous regression of the tumour has also been observed [3, 4]. However, cases of progressive growth can also be found in the literature, associated with exudative retinal detachment, intravitreal bleeding and dissemination, and with the development of neovascular glaucoma [5–8]. These cases can be treated with laser coagulation, cryotherapy or photodynamic therapy at the tumour site [9, 10]. A surgery is necessary in cases of retinal detachment. Severe neovascular glaucoma may be an indication for bulbar enucleation [5]. It is unclear whether solitary astrocytic hamartomas not associated with general symptoms of phacomatoses represent a separate clinical unit or whether they are a partial expression of tuberous sclerosis or a forme fruste [1]. Neurofibromatosis (or von Recklinghausen disease) is associated with typical discoloured café au lait skin patches and with plexiform neurofibromas in the area of eyelids and central nervous system (CNS) meningiomas [11–13]. Tuberous sclerosis (or Bourneville-Pringle disease) is associated with the ocular finding of adenoma sebaceum – butterfly-like vascularized red papulae on the skin of the nose and face or, possibly, in rare cases discoloured café au lait skin patches; astrocytic hamartoma may also develop in the CNS and cause epilepsy.

Fluorescein angiography clearly shows a network of small capillaries of the tumour in the arterial and venous phase, later accompanied by its homogeneous hyperfluorescence [1, 14], gaining a mulberry-like appearance of the surface [14]. Tumour autofluorescence depends on the range of calcification. The calcified part of the tumour shows strong autofluorescence; noncalcified parts block retinal autofluorescence [15]. Optical coherence tomography shows a hyperreflexive formation growing from the layer of retinal nerve fibres with a preserved structure of external layers of the retina and smooth transition to the surrounding normal retina. A shadow may be apparent, caused by calcifications in the tumour. The finding may also be associated with vitreous traction on the tumour surface and an edema in the area of the tumour or in the macula [16]. Ultrasound examination shows astrocytic hamartoma as an elevation at the lesion site with high reflectivity, often with calcifications. A fine-needle biopsy can be performed in the case of unclear findings [17].

As regards differential diagnosis, several additional modalities should be considered. In particular, other glial tumours of the retina are concerned – presumed solitary circumscribed retinal astrocytic proliferation (PSCRAP) and acquired retinal astrocytoma [1].

PSCRAP is found in elderly patients without a history of tuberous sclerosis or neurofibromatosis. Compared to astrocytic hamartoma, such lesions are usually well circumscribed, unilateral flat formations, shadowing deeper retinal structures. They do not include calcifications. SD-OCT scans clearly show their origin in the nerve fibres layer with the shadowing of deeper retinal layers. No edema is usually present at the lesion site or in the macula. They are stable lesions without any tendency to grow and cause complications [18].

Acquired retinal astrocytoma is found in adult patients without a history of tuberous sclerosis or neurofibromatosis [1]. It is a solitary yellowish tumour. Calcifications are not usually present. Unlike astrocytic hamartoma, the astrocytoma tends to grow slowly, which is usually associated with intraretinal exudation and secondary retinal detachment [5–8]. Our patient did not meet the criteria for any of these diagnoses.

Macular telangiectasia type 2 is a progressive bilateral disease of the macula of unknown etiology, with characteristic alterations of the macular capillary network and with the formation of parafoveolar telangiectasias; in later stages it shows dilated capillaries and retinal atrophy [2, 19]. Other characteristics include reduced transparency of the retina (greying), crystalline deposits, foveal atrophy and retinal pigment epithelium hyperplasia. Müller cell counts of the retina [20] also decrease, as well as the density of macular pigments lutein and zeaxanthin [21]. All these alterations are usually most prominent in the temporal region from the foveola, up to about 1 disc diameter, but they may also involve a circular area around the foveola. The alterations do not respect the horizontal raphe of the retina. The disease may be complicated by the development of neovascular membrane [2, 19] and macular hole [2, 22]. Prevalence of the disease in the white population has been reported between 0.004 % and 0.1 % [23–25], with the same incidence in both sexes [2]. Family incidence of the disease has been known [2, 26, 27]. Autosomal dominant inheritance is presumed with reduced penetration and variable expressivity. No specific gene responsible for the disease has been identified [27]. Several classifications of the disease have been proposed. Gass and Blodi proposed to divide the disease into 5 stages based on chronological development of alterations in the fundus [2]. Yannuzi et al. suggested dividing the disease to a nonproliferative and proliferative stage [19]. Many therapeutical approaches have been tried – laser photocoagulation of the retina [2, 28], intravitreal triamcinolone [29, 30], photodynamic therapy [31, 32], and antiVEGF intravitreal injections [33, 34], [32, 35]. Efficacy of antiVEGF products and photodynamic therapy has been shown in findings complicated by neovascular membrane [36–40]. No efficient therapy has been known yet for the nonproliferative stage of the disease [32, 41].

Concurrence of astrocytic hamartoma and telangiectasias has been described [42]. However, this was related to a unilateral finding in a patient with tuberous sclerosis. The astrocytic hamartoma was flat, diffuse, not quite clearly expressed. Neovascularizations localized in the superior region from the papilla and along the inferotemporal arcade were already present at the time of the diagnosis. Telangiectasias in the macular area, in the inferior and nasal regions from the foveola, were discovered in year 2 of the patient follow-up; hard exudates were present in the area of the telangiectasias, and FA clearly showed leakage from the telangiectasias. The finding in the patient thus does not correspond to a finding typical for macular telangiectasia type 2.

## Conclusion

Concurrent incidence of solitary astrocytic hamartoma of the retina and of macular telangiectasia type 2 was present in our patient. We could not find any evidence of any relationship between these two diseases in available literature (PubMed). In this case, it is only an accidental concurrence of two diseases given that unilateral retinal astrocytic hamartoma is combined with a bilateral finding of macular telangiectasia type 2.

## Abbreviations

CNS: Central nervous system
ETDRS: Early Treatment Diabetic Retinopathy Study charts
FA: Fluorescein angiography
PSCRAP: Presumed solitary circumscribed retinal astrocytic proliferation
SD-OCT: Spectral domain optical coherence tomography
VA: Visual acuity
